# Pgrmc1 Knockout Impairs Oocyte Maturation in Zebrafish

**DOI:** 10.3389/fendo.2018.00560

**Published:** 2018-09-24

**Authors:** Xin-Jun Wu, Peter Thomas, Yong Zhu

**Affiliations:** ^1^Department of Biology, East Carolina University, Greenville, NC, United States; ^2^Marine Science Institute, University of Texas at Austin, Port Aransas, TX, United States

**Keywords:** Pgrmc1, oocyte maturation, progestin, mPRα, Paqr7, subfertility

## Abstract

Recent investigations suggest progestin receptor membrane component 1 (PGRMC1) associates with and transports a wide range of molecules such as heme, cytochromes P450, steroids with 21 carbons, membrane progestin receptor alpha (mPRα/Paqr7), epidermal growth factor receptor (EGFR), and insulin receptor. It is difficult to discriminate the true functions of PGRMC1 from the functions of its associated molecules using biochemical and pharmacological approaches. To determine the physiological function(s) of PGRMC1, we generated global knockouts for *pgrmc1* (*pgrmc1*^−/−^) in zebrafish. We found a reduction in both spawning frequency and the number of embryos produced by female mutants. We also observed reduced sensitivity of fully-grown immature oocytes to a progestin hormone and a reduced number of oocytes undergone meiotic maturation both *in vivo* and *in vitro* in *pgrmc1*^−/−^. This reduced sensitivity to progestin corresponds well with significant reduced expression of mPRα, the receptor mainly responsible for mediating oocyte maturation and meiosis resumption in fish. The results provide *in vivo* and *in vitro* evidence for the physiological functions of Pgrmc1 in oocyte maturation and fertility, as well as a plausible molecular mechanism *via* regulation of mPRα, which in turn directly regulates oocyte maturation and affects fertility in zebrafish.

## Introduction

Progestin receptor membrane component 1 (PGRMC1) is a single transmembrane heme-binding protein containing a cytochrome b_5_ (Cytb5) motif (1, 2), which has been proposed as a progesterone binding protein in vertebrates (1, 3, 4). Various molecules such as heme, fatty acid 2-hydroxylase, cytochrome P-450 enzymes, insulin receptor, epidermal growth factor receptor (EGFR), Erbb2, and membrane progestin receptor α (mPRα, PAQR7) can interact and associate with Pgrmc1 (5–10). Elucidating the roles of PGRMC1 has been further complicated by its broad localization in various organelles and its purported role in transporting other molecules (11, 12). Untangling the roles of PGRMC1 from functions of its associated molecules requires further studies such as generating and characterizing PGRMC1 knockouts. Conditional knockout of PGRMC1 results have suggested subfertility in female mice (13). However, conditional ablation of PGRMC1 using promoter of Amhr2 (cre) mice to delete PGRMC1 resulted in the same number of pups/litter as wildtype (wt) mice, although conditional ablation using the promoter of nuclear progesterone receptor, Pgr (cre) caused a 40% reduction in the number of pups/litter. It is still unclear whether PGRMC1 affects female fertility in non-mammalian vertebrates that lack a uterus, and whether the difference in fertility observed between Amhr2- and Pgr- induced cre knockouts was due to off-target effects or inefficiency of *LoxP* site recombination frequently found in conditional knockouts.

To address these questions, we generated Pgrmc1 mutants in zebrafish (*Danio rerio*) using CRISPR/Cas9 gene editing technology, and characterized the phenotypes of the knockouts, and also examined the molecular mechanisms underlying the actions of Pgrmc1. We found reductions in the spawning frequencies in Pgrmc1 mutant females and the number of embryos produced. Expression of mPRα was significantly reduced in the Pgrmc1 knockouts, which is likely the cause, at least partially, of the attenuation of oocyte maturation in Pgrmc1 knockouts. Unless indicated otherwise, we will use P4 (progesterone), DHP (17α,20β-dihydroxy-4-pregnen-3-one) or progestin interchangeably in this manuscript because of conserved physiological functions and shared structure of these progestins.

## Materials and methods

### Animals

The zebrafish (*D. rerio*) strain used in this investigation, the Tübingen strain, was initially obtained from the Zebrafish International Resource Center, and then propagated in our laboratory at East Carolina University following previously published guidelines (14). All the animal care and use protocols were approved by Institutional Animal Care and Use Committee (IACUC) at East Carolina University.

### Design of Pgrmc1 knockout targets and preparation of Cas9 RNA and SgRNAs

**Figure 1:**
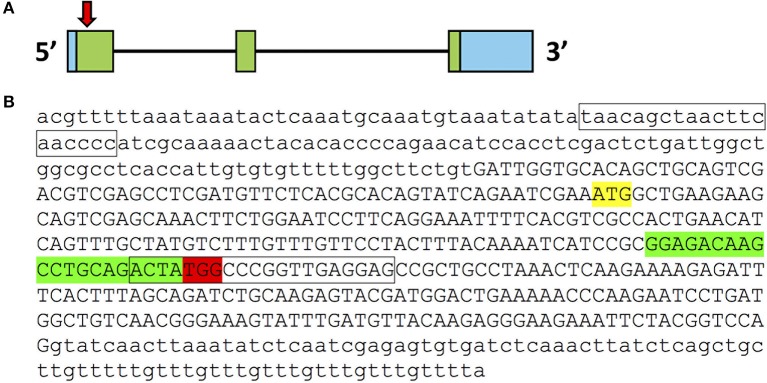
CRISPR/Cas9 target in *pgrmc1* gene. **(A)** Organization of exons and introns for *pgrmc1*. Exons are indicated by boxes; introns are indicated by black lines. Coding exons are labeled with green while untranslated regions are labeled with blue. Approximate location of the CRISPR/Cas9 target in exon 1 of *pgrmc1* is indicated by a downward red arrow. **(B)** Genomic DNA sequence of first exon (in upper case) and flanking intron regions (in lower case) of pgrmc1 are shown. Translation start sites (ATG) are highlighted in yellow. The sequences highlighted in red are PAM sequences and those in green is the CRISPR/Cas9 target site. Location of PCR primer for distinguishing the mutant from the wildtype is indicated with a box.

We identified 5′GG-(N_18_)-NGG3′ target sequences in exon 1 of *pgrmc1* (GGAGACAAGCCTGCAGACTA*TGG*) (Figure 1). Syntheses of Cas9 RNA and single guide RNAs (sgRNAs) were based on a protocol described previously (15). For synthesis of Cas9 transcripts (nls-zCas9-nls RNA), a template plasmid (pCS2-nls-zCas9-nls) was linearized by NotI digestion, then purified using a QIAprep column (Qiagen, Germantown, MD, USA). Capped Cas9 mRNA (nls-zCas9-nls) was synthesized using mMESSAGE mMACHINE SP6 kit (Fisher Scientific, Hampton, NH, USA) and purified using a RNeasy Mini kit (Qiagen). For guide RNAs (gRNAs), template plasmids were linearized by BamHI digestion and purified using a QIAprep column. The gRNAs were generated by *in vitro* transcription using a MEGAshortscript T7 kit (Fisher Scientific). The size and quality of the resulting gRNA was confirmed by electrophoresis using a 2% (wt/vol) formaldehyde agarose gel.

### Establishment of zebrafish knockout line for Pgrmc1

Screening and generation of mutant zebrafish lines for Pgrmc1 followed protocols established previously (14). To generate a founder population (F0), fertilized eggs were collected within 5 min of natural spawning of wildtype fish from their crossing tanks that were set up the night before. Microinjection was performed on newly fertilized zebrafish embryos at one or two-cell stages. Approximately 100 ng/μl of sgRNA and 150 ng/μl of Cas9 mRNA were co-injected into the embryo using a glass microcapillary pipette attached to a micromanipulator under a stereomicroscope (Leica MZ6). Injection was driven by compressed N_2_ gas, under the control of a PV820 Pneumatic PicPump (World Precision Instrument, Florida, USA). For comparison, and to estimate mutagenesis efficiency, embryos without microinjection were designated as wildtype and used as controls. A pool of genomic DNA was extracted from 30 normally developing wildtype or CRISPR/Cas9-gRNA-microinjected embryos 2-day post-fertilization (dpf) using a HotSHOT method (16). Mutation rates were estimated by comparing band intensities of undigested PCR products to intensities of digested PCR products using T7 endonucleases I assay (14). The PCR products were cleaned through a Qiagen column, cloned into a TA cloning vector, and potential mutant clones were selected for DNA sequencing analysis to confirm the presence of frame-shifting mutation (14).

To identify germline-transmitted mutations, remaining F0 founder embryos were raised to adulthood and outcrossed with wildtype fish. Genomic DNA from each cross was extracted from 30 randomly selected and pooled F1 embryos, and the status of the target site was analyzed *via* PCR amplification, T7 Endonucleases I assay, and DNA sequencing as described above. The remaining F1 embryos with identified frame shift mutations were raised to adulthood and were genotyped individually. Genomic DNA was extracted from part of the caudal fin of adult fish in a 50 μl hot alkaline solution and analyzed as stated above. Heterozygous F1 adults carrying the same frameshift mutant alleles were crossed with each other. These crosses yielded wildtype, heterozygous, and homozygous F2 fish that were further characterized genetically and physiologically. A mutant-specific reverse primer was then designed according to the mutated sequence in CRISPR/Cas9-induced mutation (*Pgrmc1*-Forward: 5′-GGGGGTGTCTGAAAGGAACT-3′ *Pgrmc1*-wildtype Reverse: 5′-CTCCTCAACCGGGCCATAGT-3′ *Pgrmc1*-mutant Reverse: 5′-GGCTCCTCAACCGAGACAAA-3′). PCR condition was optimized for efficient identification of the specific mutation. After an initial denaturation for 2 min at 94°C, the cycling reaction was performed with the profile of 30 s at 94°C, 30 s at 61°C, and 45 s at 72°C for 35 cycles, followed by a 10 min extension at 72°C with a Thermal Cycler (Eppendorf, Hauppauge, NY, USA).

### Spawning and fertility

After zebrafish reached their maturity at ~4 months of age, at least 10 homozygous mutant female fish were crossed with fertility confirmed wildtype males to check fertility of females. Production of offspring for each genotype was recorded daily for a period of 2 weeks following a 2-week acclimation period. Spawning frequency is defined as the number of times a female produce fertilized embryos in a 2-week examination period.

### Ovarian histology

Three females from each genotype were collected at 8 am (1 h before lights turned on) and deeply anesthetized in a lethal dose of MS-222 (300 mg/L buffered solution) for 10 min. To ensure death, the spinal cord and blood supply were cut off behind the gill cover using sharp scissors. Ovaries were quickly removed and examined by histology following protocols described previously (17). Briefly, fresh ovaries were fixed overnight in 10% buffered formalin (Fisher Scientific), washed in tap water, dehydrated through increased concentrations of ethanol (70, 80, 90, 100%, 30 min each) and embedded in JB-4 resin (Polysciences, Warrington, PA). Sections of 5 μm were cut and stained with hematoxylin and eosin.

### Follicle isolation and quantification

Oocyte maturation and ovulation in zebrafish typically occurs prior to the onset of (day) light, while spawning occurs within 1 h following the onset of light. Therefore, adult females (*n* = 7) from each mutant genotype were euthanized at 09:30 a.m., 30 min after laboratory lights were turned on, by placing each fish in a lethal dose of MS-222 (300 mg/L buffered solution) for 10 min, then severing the spinal cord and blood supply using IACUC approved procedures. The ovaries of each fish were then immediately dissected out and rinsed in 60% L-15 media (Sigma-Aldrich, St. Louis, MO, USA) containing 15 mM HEPES (pH = 7.2). We use the term “follicles” to mean follicular cells and their enclosed oocytes in this manuscript. Follicles of various sizes were isolated from the ovaries using fine forceps. The diameter of each follicle was measured under a stereo microscope (SZX7, Olympus, Japan), and recorded. The developmental stages of follicles were divided into five stages based on morphological criteria and on physiological and biochemical events (18, 19) with a slight modification: Stage I (<140 μm) and II (140–340 μm) previtellogenic follicles; Stage III early vitellogenic follicles (340–690 μm); Stage IV late vitellogenic follicles (690–730 μm) that are further divided into IVa and IVb two stages, IVa follicles are maturationally competent fully grown immature follicles (IVa), IVb follicles are mature follicles that have undergone oocyte maturation but have not ovulated (IVb); and Stage V ovulated follicles are ovulated eggs with no follicle cells attached (730–750 μm).

### *In vitro* oocyte maturation assay

Zebrafish ovarian follicles were isolated and incubated to determine the sensitivities of follicles to a maturation inducing steroid, DHP (20, 21). Gravid female zebrafish were euthanized humanely and the ovaries were dissected out and then washed several times in 60% Leibovitz L-15 medium (Sigma-Aldrich). Individual ovarian follicles were carefully separated without damaging the follicular cell layers following previously established protocols (21, 22). Thirty follicles of the same size (follicular diameter of 550–650 μm) were selected and randomly distributed into the wells of a 24-well plate (~30 follicles/1 ml medium/well), and then treated with 1 μl DHP dissolved in ethanol. Final concentrations for DHP and ethanol in the incubation medium were 5 nM and 0.1%, respectively. As controls, 1 μl ethanol was added into the control wells containing the same number of follicles (~30 follicles/1 ml medium/well) collected at same time from the same group of individual fish, and the rates of GVBD were also recorded. Incubation of ovarian follicles was continued for up to 5 h, with GVBD being scored each half hour during the incubation period. All experiments were repeated five times to confirm the results.

### Membrane receptor binding assay

Membrane binding of progesterone were conducted as described previously (23) using [1,2,6,7-^3H^] progesterone (85 Ci/mmol, American Radiolabeled Chemicals, St. Louis, MO, USA). Plasma membrane fractions of ovaries were obtained following established procedures with few modifications (24, 25). The ovaries from wildtype or Pgrmc1 knockout fish were washed with assay buffer and then sonicated for 15 s, followed by a 1,000 g centrifugation for 7 min to remove any nuclear and heavy mitochondrial material. The resulting supernatant was centrifuged at 20,000 g for 20 min to obtain the plasma membrane fraction. Progesterone receptor binding in the membrane fractions was measured in a single-point assay as described previously (26). One set of tubes contained radiolabeled progesterone alone (final concentration: 2.6 nM, total binding), another set also contained cold progesterone competitor at a 400-fold greater concentration (1 μM) to measure non-specific binding. After a 30-min incubation at 4°C with the membrane fractions (160 μg membrane protein), the reaction was stopped by filtration (Whatman GF/B filters, presoaked in assay buffer; Fisher Scientific, Pittsburgh, PA, USA). The filters were washed twice with 25 ml assay buffer and the bound radioactivity was measured by scintillation counting. The results were expressed as DPM specific binding of [3H]-progesterone.

### RNA isolation and real-time quantitative PCR

Total RNA was isolated from various tissues using the RNAzol reagent (Molecular Research Center, Cincinnati, OH, USA) according to a modified protocol (22), and reverse transcribed to cDNA using a High Capacity cDNA Reverse Transcription kit (Thermo Fisher). The amount and purity of the RNA was determined using a Nanodrop 2000 (Thermo Fisher). Real-time quantitative PCR (qPCR) was performed using SYBR green with a C1000 Touch Thermal Cycler (Bio-Rad). PCR efficiency calculated from the equation of efficiency (EFF) = 10(−1/slope) −1 and authentic PCR products were confirmed by analyses of melting curves, gel electrophoresis, and DNA sequencing. PCR data were analyzed using the absolute quantitation method, expressed as copies/μg total RNA, and were determined using Ct values of samples and a standard curve from serial known concentrations of plasmids containing different cDNA fragment of target genes. The full names of the genes and the primers used in this study are listed in Table 1.

**Table 1:** Real time quantitative PCR (qPCR) primers used in this study.

**Name**	**Strand**	**Sequences (5′-3′)**	**Product size (bp)**
*pgrmc1*	F	CAGACTATGGCCCGGTTGAGGAG	281
	R	CTGCATGGCATTGAGATCGG	
*mPRα*	F	CGTACTTGTGAGTGCAAGGGT	165
	R	GAAACGAGGGATCTGGCGAA	
*pgr*	F	ACAGACAGCATACACCGC	103
	R	TCCACAGGTCAGAACTCC	
*lhcgr*	F	CGCTCTGATCAACTGGGACA	218
	R	GGCGCTGTTGGCATAAATCC	
*adam8b*	F	CCTGGCATCCACAATTGCAC	254
	R	CATTACCACAGACAGGCCCA	

### Western blotting

Expression of mPRα, the nuclear progesterone receptor (Pgr), and Pgrmc1 in the fully-grown Stage IVa immature follicles was confirmed by Western blot analysis using previously characterized polyclonal zebrafish antibodies (8, 21, 27). In brief, Stage IVa follicles were collected from adult zebrafish following protocols described previously (21, 22). Ten Stage IVa follicles collected from freshly sacrificed fish were sonicated in 100 μl of 1x SDS sample buffer (62.5 mM Tris-Cl pH 6.8, 2% SDS, 10% glycerol, 100 mM dithiothreitol) on ice for about 10 short bursts (Sonic Dismembrator, Fisher Scientific). Protein samples were then immediately boiled for 10 min and stored in at −20°C until analysis. Electrophoresis was conducted with 10 μl of each sample (~one follicle) per lane on a 8% SDS PAGE gel (12% for Pgrmc1), and the resulting protein bands were transferred to a nitrocellulose membrane. The membrane was first pre-incubated for 3 h with a blocking solution containing 5% BSA (albumin from bovine serum, Sigma A7906) in TBST (50 mM Tris, 100 mM NaCl, 0.1% Tween 20, pH 7.4). The samples were then incubated with the primary antibodies (mPRα, 1:250 dilution; Pgr, 1:250 dilution; Pgrmc1, 1:250 dilution; α-tubulin (Sigma, T6074), 1:3,000 dilution) in the 1% BSA blocking solution overnight. The following day, the membrane was washed five times for a period of 5 min each with 1x TBST, incubated for 2 h with horseradish peroxidase conjugated secondary antibody (1:5,000 dilutions, goat anti-rabbit antibody for mPRα, Pgr, and Pgrmc1 detection or goat anti-mouse antibody for α-Tubulin), and finally washed five times for a period of 5 min each with 1x TBST. The membranes were developed using Super Signal West Extended Dura Substrate (Pierce, Rockford, IL, USA) in a plastic wrap, then visualized using a Fluor Chem 8900 imaging station (Alpha Innotech, San Leandro, CA, USA). Protein size was determined by comparison to a biotinylated protein ladder (Cell Signaling Technology, Danvers, MA, USA) and a prestained protein ladder (Fermentas). Finally, image analyzing software (ImageJ) was used to estimate relative densitometries (28) and normalized using expression of α-tubulin.

### Statistical analysis

All the results were analyzed using GraphPad Prism 7.0a (San Diego, CA, US) and presented as mean ± SEM. Significant differences among paired treatment groups were determined using Student's *t*-test. Gene expressions at different time points and tissues were analyzed by one-way analysis of variance (ANOVA). Using two-way ANOVA to compare the differences between follicular cells and denuded oocytes over different time points followed by Tukey's multiple comparisons test. Statistical significance was set at *P* < 0.05.

## Results

### Establishment of *pgrmc1* knockout lines in zebrafish

**Figure 2:**
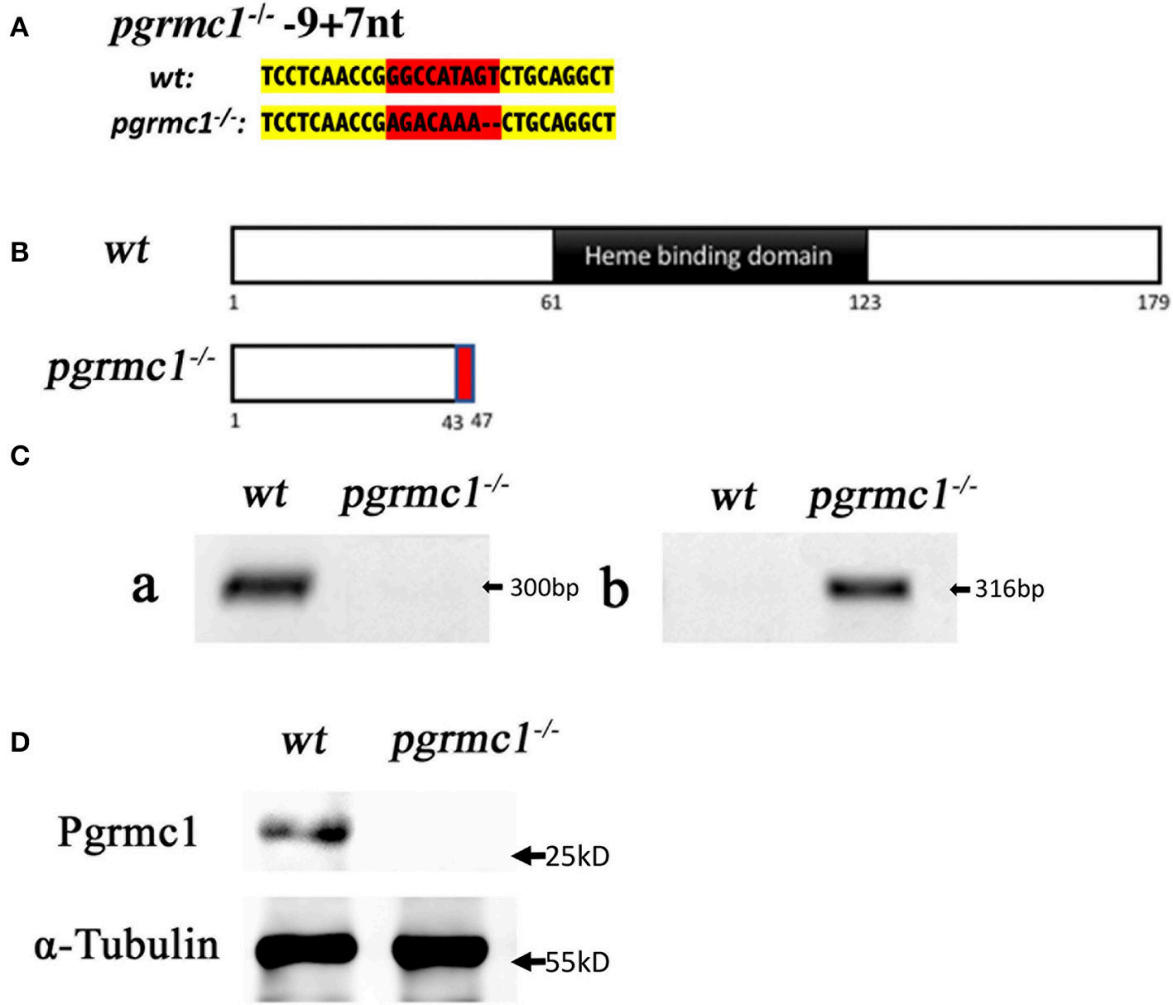
Gene editing of *pgrmc1* in zebrafish. **(A)** Comparison of mutant genomic DNA sequences (small insertions or deletions) in *pgrmc1*^−/−^ to those in wildtype (*wt*). Modified sequence region is highlighted in red, whereas adjacent un-modified sequences are highlighted in yellow. **(B)** Schematic drawings show *wt* Pgrmc1 protein and predicated truncated proteins from *pgrmc1*^−/−^ lacking heme binding domains. **(C)** Gel images of PCR products using a *pgrmc1* wildtype specific primer (a), or a *pgrmc1* mutant specific primer (b) to distinguish Pgrmc1 mutants from wildtype fish. **(D)** Western analysis confirmed loss of Pgrmc1 protein in *pgrmc1*^−/−^ using Stage IVa follicles and a zebrafish Pgrmc1 specific antibody.

We targeted the first exon of *pgrmc1* as it contains the proper parameters for CRISPR/Cas9 target design (Figure 1). Successful gene editing by CRISPR was confirmed by Sanger sequencing. Several mutant lines were established; a representative line is described below. One mutant line had a deletion of 9 nucleotides and an insertion of 7 nucleotides in the *pgrmc1* gene, which led to a frame shift mutation, a premature stop codon and a truncated protein (Figures 2A,B). This CRISPR/Cas9-gRNA-induced specific deletion and insertion effectively disrupted the translation of *pgrmc1*, with the loss of heme binding domain (Figure 2B). Using specific primers that target a frame shift site, we could easily differentiate *pgrmc1*^−/−^ from *wt* using PCR (Figure 2C). Loss of protein was confirmed by Western analysis using a specific antibody against zebrafish Pgrmc1 (Figure 2D).

### Reduced fertility in *pgrmc1^−/−^* female zebrafish

**Figure 3:**
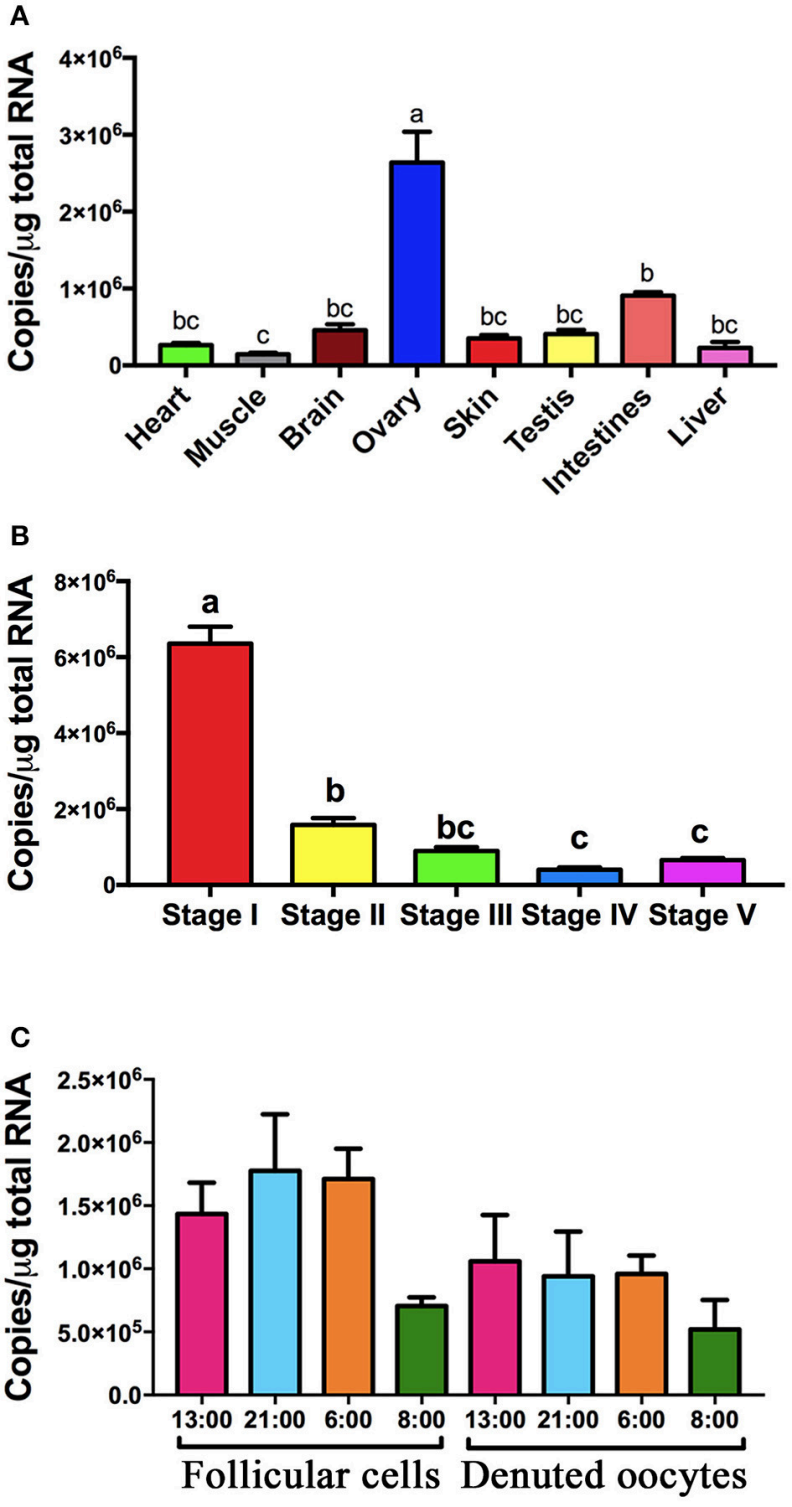
Expression of *pgrmc1* in zebrafish. **(A)** Expression of *pgrmc1* in various tissue types. **(B)** Expression of *pgrmc1* in different stages of follicles. **(C)** Expression of *pgrmc1* in follicular cells and denuded oocytes. Bars with different letters indicated significant difference.

Expression of *pgrmc1* was found in all tissues examined, including all reproductive tissues and all stages of follicles with highest expression in the ovaries and in Stage I follicles (Figures 3A,B). Expression of *pgrmc1* were also found in both follicular cells and denuded oocytes of Stage IV follicles with no significant difference among samples collected at different times of day (Figure 3C).

**Figure 4:**
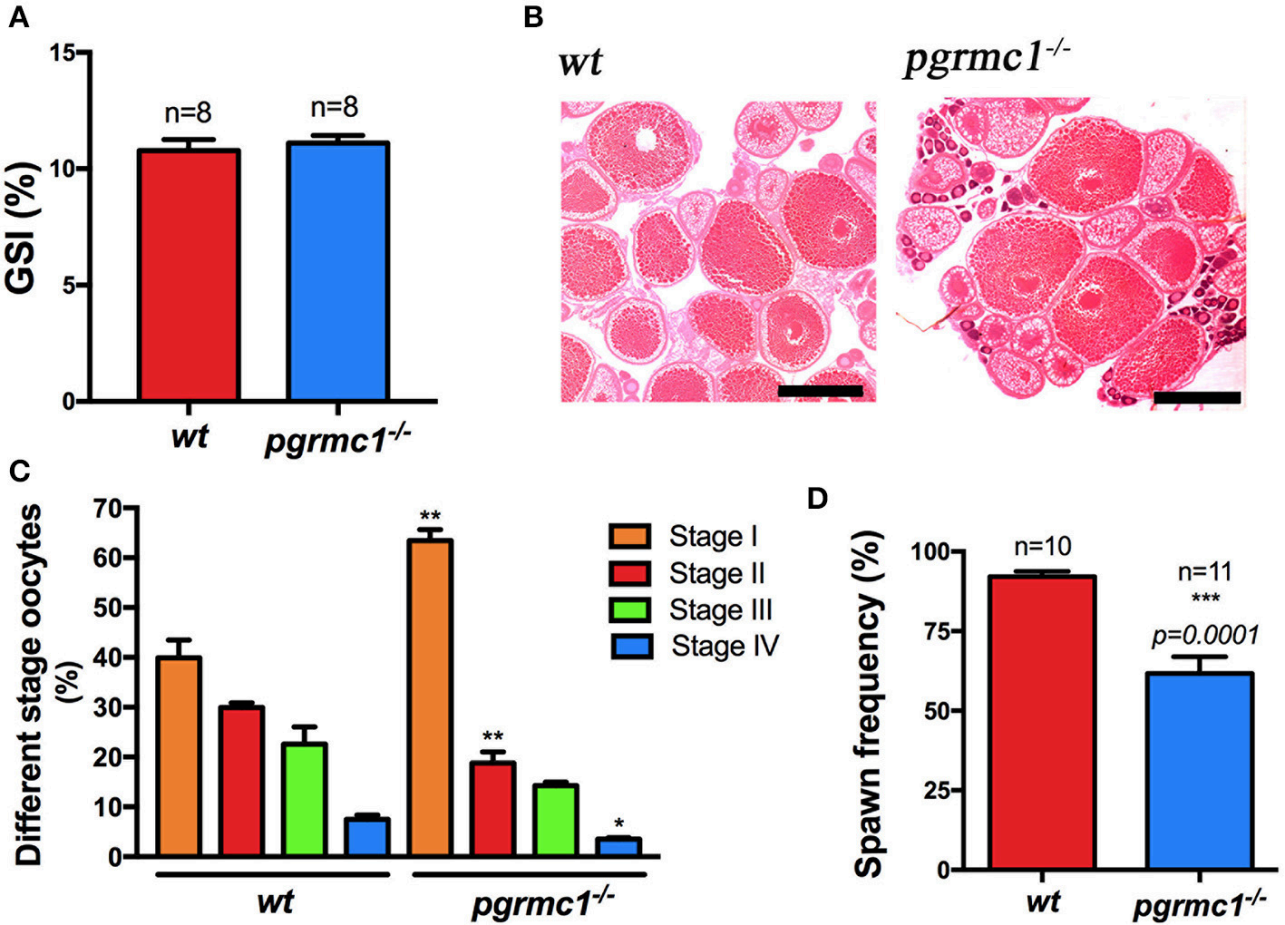
Comparison of ovarian size, morphology and spawning frequency between *pgrmc1*^−/−^ and wildtype. **(A)** Gonadosomatic index (GSI) of wildtype (*wt*) and *pgrmc1*^−/−^. **(B)** HE staining of a representative ovarian section from a *pgrmc1*^−/−^ female showed well-formed different stages of oocytes with a significantly higher number of Stage I follicles compared to wildtype (*wt*). Scale bars: 500 μm. **(C)** A higher number of Stage I follicles but a lower number of late stage follicles were observed in *pgrmc1*^−/−^ compared to *wt*. **(D)** Mutant females spawned with less frequency than *wt*. ^*^p < 0.05; ^**^p < 0.01; ^***^p < 0.001.

Anatomical examination revealed no obvious differences in overall ovarian size (GSI, i.e., gonadal somatic index, Figure 4A) and oocyte growth or development (Figure 4B) between *pgrmc1*^−/−^ females compared with their wildtype siblings, although more early stage (Stage I) follicles found in *pgrmc1*^−/−^ (Figure 4C). To further evaluate the reproductive capacities in mutant zebrafish, mature *pgrmc1*^−/−^ females (at least 10 from each genotype) at 4 months of age were mated with known fertile wildtype males during a minimum 4-week mating studying period. The fecundity of mutant zebrafish, determined as spawning frequency, and total number of embryos produced over 2 weeks following a 2-week acclimation period, was recorded and compared to those in wildtype crossing (wildtype males crossed with wildtype females) that were treated the same during same period. Pgrmc1 mutant females spawned with significantly less frequency (*n* = 11, 61.69 ± 5.30%) compared to wildtype females (*n* = 10, 92.14 ± 1.67%) (Figure 4D).

**Figure 5:**
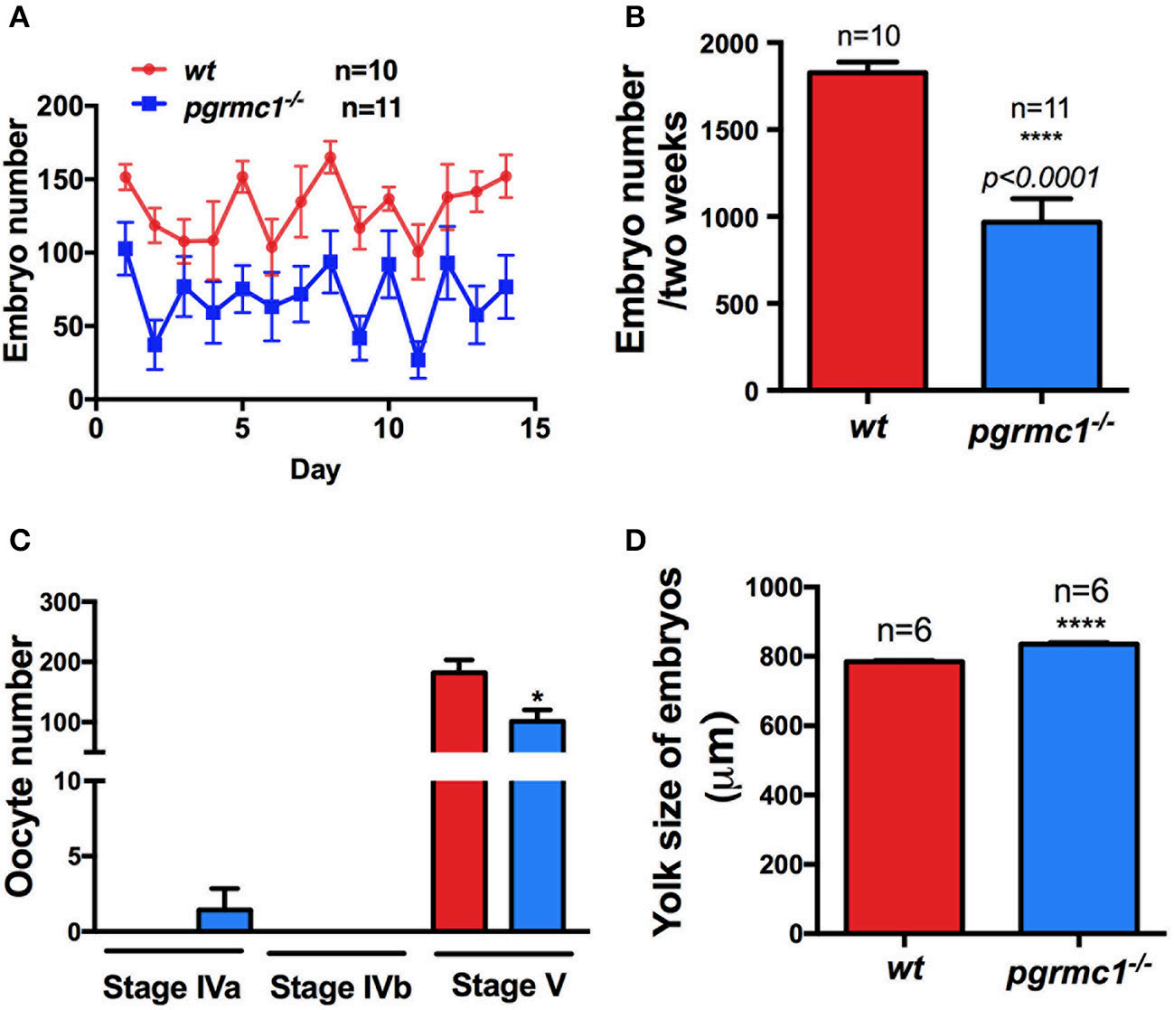
Reduced fertility in *pgrmc1*^−/−^ female zebrafish. **(A)** Mutant females produced fewer embryos daily than wildtype (*wt*) females. **(B)** Mutant female zebrafish produced fewer embryos over a 2-week mating period. **(C)** Some leftover immature Stage IVa oocytes were observed in *pgrmc1*^−/−^, while none were leftover in *wt* as all these oocytes had completed maturation and ovulation before lights turned on in the morning. Consequently, fewer ovulated Stage V oocytes were found in *pgrmc1*^−/−^. **(D)** Significantly larger yolks were observed in *pgrmc1*^−/−^ embryos in comparison to those from *wt*. ^*^p < 0.05; ^****^p < 0.0001.

Interestingly, *pgrmc1*^−/−^ females produced a significantly lower number of live embryos daily than wildtype females over 2-week continuous mating tests (Figures 5A,B; *pgrmc1*^−/−^: 968 ± 134.8 live embryos per female in 2 weeks; *wt*: 1827 ± 63.2 live embryos per female in 2 weeks, *p* < *0.0001*). The lower number of live embryos produced by mutants was not due to reduced survival of embryos since similar survival rates were observed in *pgrmc1*^−/−^ as in wildtype embryos (data not shown). Intriguingly, fewer follicles in *pgrmc1*^−/−^ females ovulated (Figure 5C), which is consistent with the lower number of live embryos produced by the mutants. Fewer ovulated oocytes could be due to defects in oogenesis, follicular growth or development. We observed that numbers and percentages of Stages I follicles were significantly higher, whereas percentages of Stage II to IV follicles were significantly lower in *pgrmc1*^−/−^ compared to wildtype females (Figures 4B,C). These defects might contribute to low fertility in *pgrmc1*^−/−^ female zebrafish. Typically, fully grown immature follicles (Stage IVa) would have already successfully completed the processes of oocyte maturation and ovulation after the lights had been on for half an hour, and no Stage IVa follicles would be found in the ovaries from wildtype females (Figure 5C). However, a significant number of these Stage IVa immature fully grown follicles could still be observed in the *pgrmc1*^−/−^ (Figure 5C). These results indicate that the oogenesis and oocyte maturation process *in vivo* was affected in *pgrmc1*^−/−^. Attenuated oocyte maturation also affected embryo sizes. The yolk size of each genotype was determined in sphere to 30% epiboly stage embryos. As predicted, the mean yolk size of *pgrmc1*^−/−^ embryos was larger than that of wildtype individuals (Figure 5D), likely caused by delays in the maturation process.

### Reduced oocyte maturation and expression of mPRα in *pgrmc1*^−/−^

**Figure 6:**
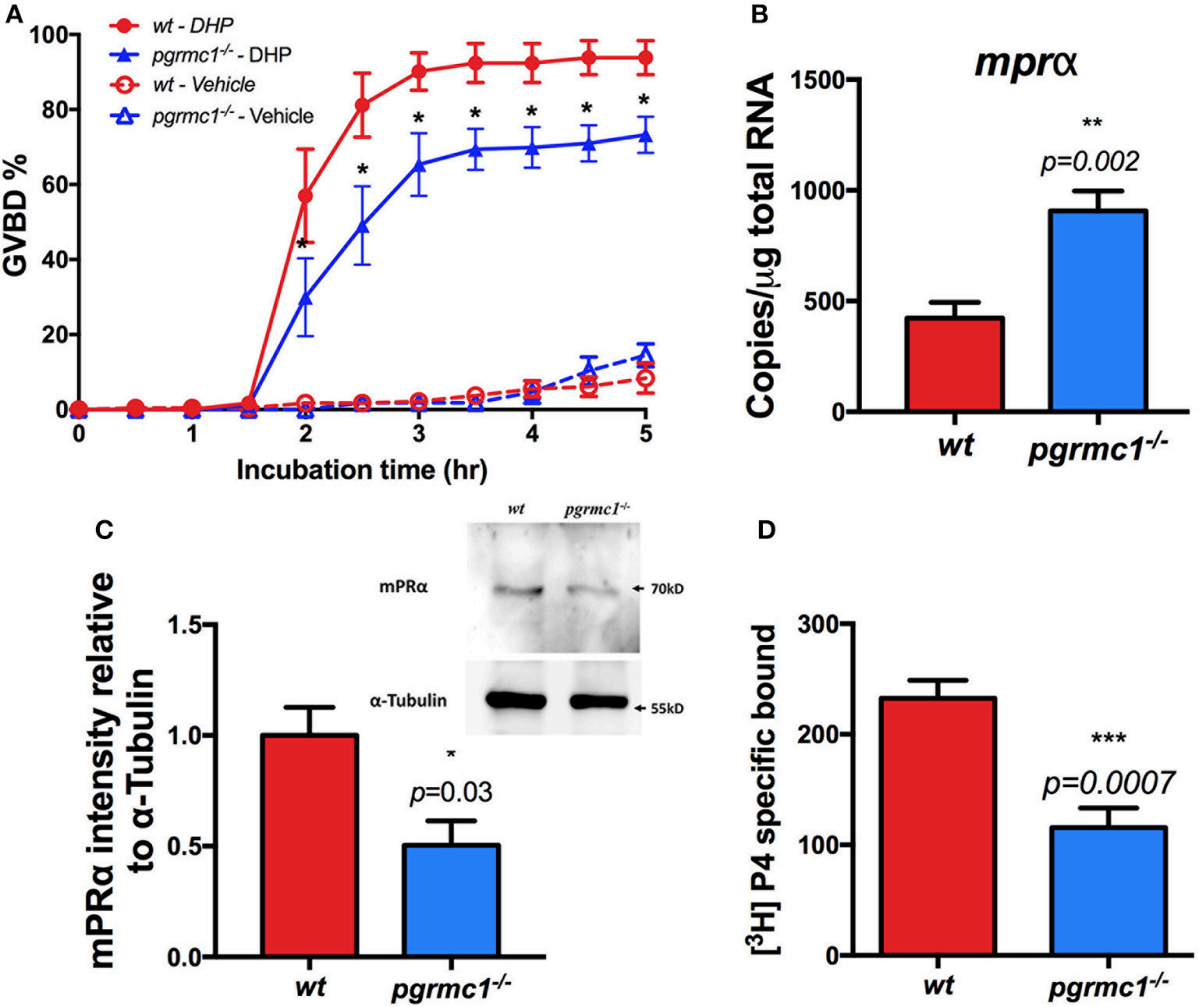
Attenuation of oocyte maturation and reduced mPRα expression in *pgrmc1*^−/−^. **(A)** Reduced sensitivity and oocyte maturation in response to progestin (DHP, 17α,20β-dihydroxy-4-pregnen-3-one) stimulation *in vitro* in Stage IVa fully-grown immature follicles from *pgrmc1*^−/−^. **(B)** Transcript of *mPR*α was upregulated in Stage IVa follicles in *pgrmc1*^−/−^. **(C)** Significant low level of mPRα protein in Stage IVa follicles in *pgrmc1*^−/−^ (*n* = 4). Insert, a representative Western blot of proteins extracted from Stage IVa follicles. **(D)** Lower progesterone binding was found in *pgrmc1*^−/−^ ovarian membrane compared with wildtype. *n* = 6. ^*^p < 0.05; ^**^p < 0.01; ^***^p < 0.001.

To examine whether the attenuated oocyte maturation is due to down-regulation of sensitivity to progestin in the Stage IVa follicles, *in vitro* GVBD tests were conducted. A slower rate of oocyte maturation and a significant decrease in the percentage of follicles that underwent maturation in response to DHP was observed in *pgrmc1*^−/−^ compared to those in wildtype (Figure 6A).

To further elucidate the molecular mechanisms underlying the impaired oocyte maturation in the ovaries of *pgrmc1*^−/−^, the expression of maturation related genes, including mPRα, was analyzed. We found reduced expression of the mPRα protein in Stage IVa follicles collected from *pgrmc1*^−/−^ (Figure 6C), although the transcripts of mPRα were upregulated (Figure 6B). No significant difference was found in expression of other related genes including mPRβ (Paqr8), mPRγ1 (Paqr5a), and mPRδ (Paqr6) in Stage IVa follicles between *pgrmc1*^−/−^ and wildtype (data not shown). Expression of mPRγ2 (Paqr5b) or mPRε (Paqr9) were below the qPCR detection limit following 45 PCR cycles. Moreover, lower progesterone binding was found in ovarian membranes obtained from *pgrmc1*^−/−^ compared to wildtype (Figure 6D).

### Oocyte ovulation process was not affected in *Pgrmc1^−/−^*

**Figure 7:**
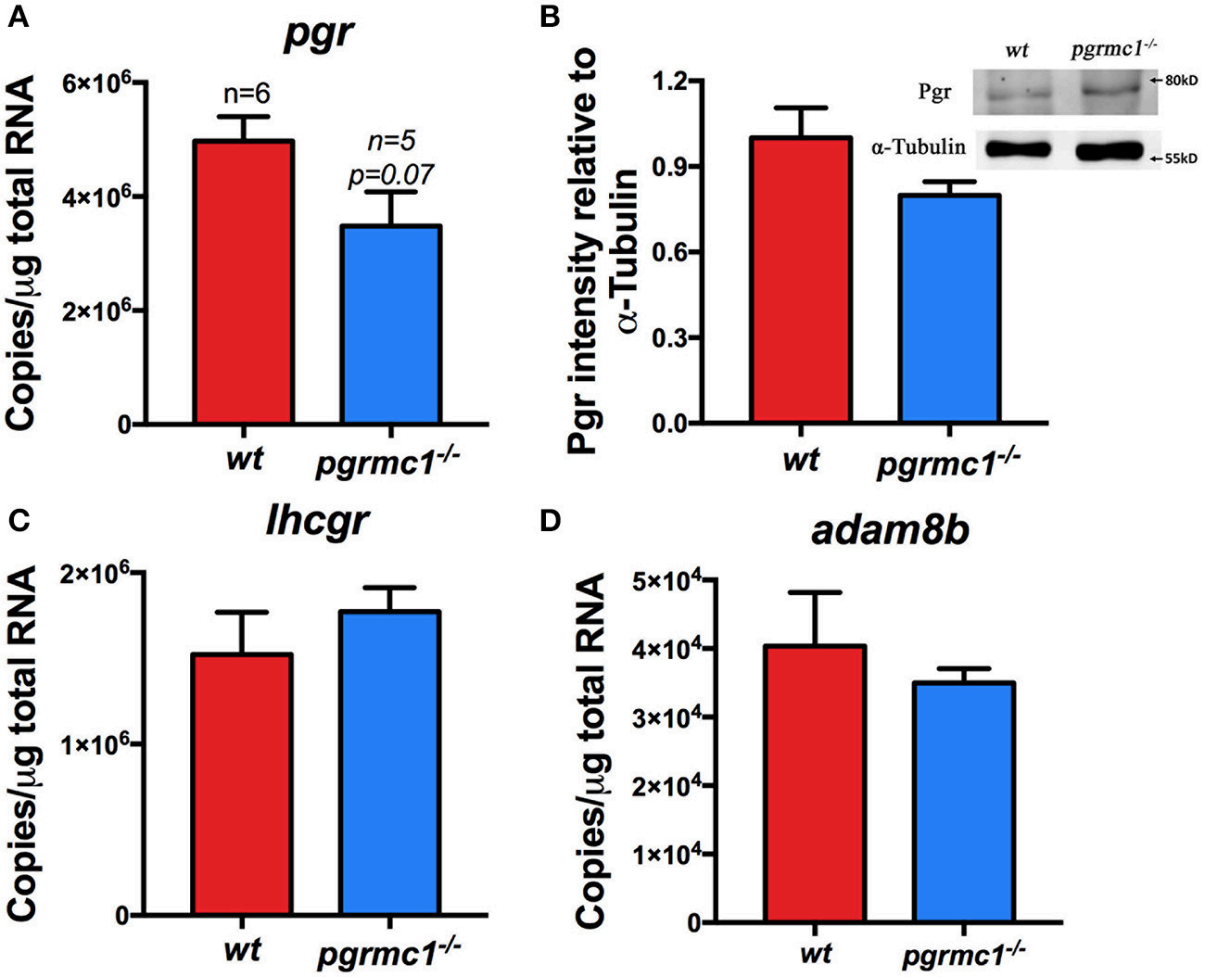
No significant difference in the expression of *pgr, lhcgr*, and *adam8b*. **(A)** Transcript of *pgr* in Stage IVa follicles in *pgrmc1*^−/−^ and wildtype (*wt*). **(B)** Pgr protein in the Stage IVa follicles of *pgrmc1*^−/−^ and *wt*. Insert, a representative Western blot of proteins extracted from Stage IVa follicles from *pgrmc1*^−/−^ or *wt*. **(C,D)** Transcripts of *lhcgr* or *adam8b* in Stage IVa follicles in *pgrmc1*^−/−^ or *wt* (*n* = 6).

Mature, but not ovulated, follicles (Stage IVb follicles) could not be found in the ovaries of *wt* and *pgrmc1*^−/−^ *in vivo* 30 min after daily lights were turned on (Figure 5C). This indicates the oocyte ovulation process was not affected in mutants. Transcript and protein levels of Pgr, a master regulator of ovulation, were not significantly different than in *wt* and *pgrmc1*^−/−^ Stage IVa oocytes (Figures 7A,B). The expression of other genes involved in ovulation, such as luteinizing hormone/choriogonadotropin receptor (*lhcgr*) and ADAM metallopeptidase domain 8b (*adam8b*) were also not affected in *pgrmc1*^−/−^ (Figures 7C,D).

## Discussion

In female vertebrates, progestin signaling is essential for gonadal development, follicular apoptosis, oogenesis, oocyte maturation, and ovulation (29–32). The classical (genomic) mechanism of progestin action mediated through a transcription factor, the nuclear receptor, Pgr, has been extensively investigated and well characterized (14, 33–35). In addition, it has become widely recognized that progestins and other steroid hormones also exert non-classical actions involving activation of intracellular signaling pathways and a rapid cellular response which is often non-genomic. However, the identities of the receptors mediating these rapid progestin actions are still the subject of active debate (30, 31, 36). To date, three candidate families of progestin receptors have been suggested to have various roles related to non-classical progestin signaling and functions, Pgr in an extra-nuclear location (37), membrane progesterone receptors [mPRs/PAQRs, (25, 26)], and members of the PGRMC family (8, 13, 30, 31, 36, 38–45). On the other hand, cumulative evidence has indicated that PGRMC1 has numerous other functions (36, 42, 43). For example, PGRMC1 is a heme-binding protein with a cytochrome b_5_ motif, which binds and activates P450 proteins and is involved in steroidogenesis and drug metabolism (2, 6, 7, 42, 46–49). Pgrmc1 has also been suggested to be an adaptor protein which facilitates membrane localization of membrane receptors, and associates with various signaling molecules at the plasma membrane (5, 8, 10, 40, 45, 50, 51). It is difficult to distinguish the functions of PGRMC1 from the functions the molecules it is associated with based on traditional biochemical or pharmacological approaches. To circumvent this problem, we generated Pgrmc1 zebrafish knockouts, and found relatively normal development and growth of follicles with more Stage I but fewer Stage V follicles (ovulated follicles) in *pgrmc1*^−/−^ compared with those in wildtype. Our result indicates that Pgrmc1 has important role in the oogenesis and late stages of oocyte development. One possible mechanism is *via* regulation of mPRα expression in the plasma membrane of follicles, which in turn regulates final oocyte maturation in zebrafish (21, 25). Mice with Pgrmc1 conditional knockout also had fewer antral follicles (13, 32). In summary, Pgrmc1 has a conserved role in the regulation of follicle development in females.

Our *in vivo* and *in vitro* results showed that maturation of fully grown immature oocytes was significantly delayed in *pgrmc1*^−/−^ zebrafish. Using biochemical and pharmacological approaches, a similar decrease in progestin (DHP) induced maturation was obtained when a Pgrmc1 inhibitor, AG205, was used in an *in vitro* zebrafish oocyte maturation assay (8, 45). Pgrmc1 is unlikely to directly regulate oocyte maturation, as there is no evidence for a direct interaction between Pgrmc1 and the inhibitory G-protein known to be important for meiosis resumption in fishes. There is now extensive evidence in several teleost models that progestin induction of oocyte maturation is dependent upon a gonadotropin-dependent increase in oocyte membrane expression of mPRα (8, 25, 31, 52, 53). Therefore, Pgrmc1 likely facilitates oocyte maturation through regulating the plasma membrane localization of mPRα. Pgrmc1 co-immunoprecipitates with mPRα, and manipulation of Pgrmc1 expression with siRNA or mRNA transfection alters the plasma membrane localization of mPRα in breast cancer cells and in zebrafish oocytes resulting in alterations in mPRα functions (8, 40). Our recent study also indicates a close association between Pgrmc1 and mPRα, and between Pgrmc1 and Erbb2 in zebrafish oocytes at the plasma membrane of Stage IV follicles using *in situ* proximity ligation assays (8). Taken together, these results suggest that Pgrmc1 can regulate the expression and localization of proteins such as mPRα at multiple levels including transcription regulation, membrane localization, and signaling of mPRα, which in turn controls oocyte maturation. Besides mPRα, Pgrmc1 has also been suggested to be associated with chromatin and to colocalize with Aurora kinase B (Aurkb) during metaphase I and II (54, 55).

The mechanism through which Pgrmc1 regulates mPRα protein expression in zebrafish ovarian remains unclear from the present results. One possible consequence of coupling mPRα to Pgrmc1 is that degradation of the mPRα protein is reduced, perhaps through a decrease in the covalent attachment of ubiquitin, resulting in maintenance of physiological intracellular mPRα concentrations. Such a mechanism would explain the apparent discrepancy in the expression mPRα mRNA and protein levels in *pgrmc1*^−/−^ follicles where mPRα protein levels are decreased without a concomitant reduction in mPRα mRNA expression which instead shows a compensatory increase. This potential additional role of Pgrmc1 in regulating the functions of proteins through controlling their degradation rate warrants further investigation.

Pgrmc1 was also been suggested to be involved in estrogen maintenance of zebrafish oocyte meiotic arrest, *via* regulating G protein-coupled estrogen receptor 1 (Gper) -dependent epidermal growth factor receptor (Egfr) signaling (45). The Pgrmc1 inhibitor, AG205, blocks the stimulatory effect of an Egfr inhibitor in oocyte maturation, and decreases expression of Egfr on the oocyte membrane (45). Pgrmc1 may inhibit or stimulate oocyte maturation by acting as an adaptor protein involved in membrane trafficking of mPRα, Egfr, or Erbb2 according to development stage of the oocytes and/or serum concentrations of estrogens and progestins.

It had been suggested that PGRMC1 might compensate loss of PGR based on higher levels of the PGRMC1 transcript found in the brains of PGR-KO female mice (56). However, no changes of PGR was observed in the ovaries of PGRMC1 knockout mice (13). Similarly, we did not find any significant difference in the expression of Pgr protein or transcript in Stage IVa follicles in *pgrmc1*^−/−^ in comparison with those in wildtype zebrafish, as well as the ovulation process in *pgrmc1*^−/−^.

Pgrmc1 has numerous actions and is broadly distributed in zebrafish tissues and throughout the brain-pituitary-gonad axis in mammals (57). Therefore, it is likely that the Pgrmc1 knockout caused defects in physiological processes other than oocyte maturation, which may contribute to the subfertility observed. Pgrmc1 has a role in the regulation of cholesterol and steroid synthesis because of its cytochrome b_5_ motif and its ability to bind and activate P450 proteins (7, 58). Knocking out Pgrmc1 might cause deficiencies in cholesterol synthesis and increased apoptosis, which could lead to reduced fertility (59, 60). Pgrmc1 has also been suggested to play a role in the conversion of testosterone into estrogen (61). Estrogen is important for vitellogenesis and growth of oocytes. Knocking out Pgrmc1 may lead to low levels of estrogen in mutants, which in turn may contribute to slow growth of oocytes and subfertility. Interestingly, we found significant high percentage of Stage I oocytes in Pgrmc1 knockout zebrafish, which could be due to defects of estrogen production. Pgrmc1 also likely has important reproductive functions in organs other than ovaries, such as in the hypothalamus where rapid effects of progestins on gonadotropin releasing hormone (GnRH) secretion have been demonstrated in fish (52). There is evidence that PGR-independent rapid progestin effects in rodent GnRH-secreting cells are mediated through mPRα and mPRβ (44), Therefore, Pgrmc1 could potentially alter GnRH secretion through regulating the membrane expression of mPRs on GnRH-secreting cells. A possible deficiency in the synthesis and release of GnRH in the zebrafish brain induced by a Pgrmc1 deficit in the knockouts might also be a cause of subfertility. However, additional research will be required to test this possibility. Nonetheless, results of our *in vitro* study suggest the expression of mPRα at the surface of oocytes regulated by Pgrmc1 is one important mechanism for Pgrmc1 regulation of female fertility.

In summary, Pgrmc1 is important for female fertility. Knocking out Pgrmc1 reduced fertility in female zebrafish. Reproductive defects in Pgrmc1 knockouts include impaired oogenesis, oocyte growth, and oocyte maturation. Our knockout also provides a model for studying functions of Pgrmc1 and its interactions with other molecules in other physiological process.

## Author contributions

YZ conceived the project and generated knockouts. X-JW and YZ designed the experiments, performed the experiments, and analyzed the data, and wrote the manuscript. PT conducted receptor binding study, contributed to the discussion and writing.

### Conflict of interest statement

The authors declare that the research was conducted in the absence of any commercial or financial relationships that could be construed as a potential conflict of interest.
